# Childbirth experience questionnaire: validating its use in the United Kingdom

**DOI:** 10.1186/s12884-015-0513-4

**Published:** 2015-04-07

**Authors:** Kate F Walker, Philippa Wilson, George J Bugg, Anna Dencker, Jim G Thornton

**Affiliations:** Maternity Department, Nottingham City Hospital, Nottingham University Hospitals NHS Trust, Nottingham, NG5 1 PB UK; Maternity Department, Queen’s Medical Centre, Nottingham University Hospitals NHS Trust, Nottingham, NG7 2UH UK; Centre for Person-centred Care, Institute of Health and Care Sciences, Sahlgrenska Academy, University of Gothenburg, Gothenburg, Sweden; Division of Obstetrics and Gynaecology, University of Nottingham, Maternity Department, Nottingham City Hospital, Nottingham, NG5 1 PB UK

**Keywords:** Childbirth experience questionnaire, Content validity, Criterion validity, Construct validity, Test-retest reliability, Birth satisfaction

## Abstract

**Background:**

The Childbirth Experience Questionnaire (CEQ) was developed in Sweden in 2010 and validated in 920 primiparous women. It has not been validated in the United Kingdom (UK).

Measuring the impact of an intervention on a woman’s childbirth experience is arguably as important as measuring its impact on outcomes such as caesarean delivery and perinatal morbidity or mortality and yet surprisingly it is rarely done. The lack of a robust validated tool for evaluating labour experience in the UK is a topical issue in the UK at present. Indeed NICE say ‘A standardised method to measure and quantify women's psychological and emotional wellbeing and their birth experiences is urgently required to support any study investigating the effectiveness of interventions, techniques or strategies during birth.’

**Methods:**

The Childbirth Experience Questionnaire and part of the Care Quality Commission Maternity Survey (2010) was sent to 350 women at one month postnatal. The CEQ was sent again two weeks later. The CEQ was tested for face validity among 25 postnatal mothers. Demographic data and delivery data was used to establish construct validity of the CEQ using the method of known-groups validation. The results of the scored CEQ sent out twice were used to measure test-retest reliability of the CEQ by calculating the quadratic weighted index of agreement between the two scores. Criterion validity was measured by calculating the Pearson correlation coefficient for the CEQ and Maternity Survey scores.

**Results:**

Face validity of the CEQ in a UK population was demonstrated with all respondents stating it was easy to understand and complete. A statistically significantly higher CEQ score for subgroups of women known to report a better birth outcome demonstrated construct validity of the CEQ. A weighted kappa of 0.68 demonstrated test-retest reliability of the CEQ. A Pearson correlation co-efficient of 0.73 demonstrated a strong correlation between the results of the CEQ and the results of the ‘gold standard’ assessment of childbirth experience in the UK: the Maternity Survey and hence criterion validity of the CEQ.

**Conclusions:**

The Childbirth Experience Questionnaire is a valid and reliable measure of childbirth experience in the UK population.

## Background

The Childbirth Experience Questionnaire (CEQ) was developed in Sweden in 2010 and validated in 920 primiparous women [1]. It measures 4 main domains of the childbirth experience: *Own capacity, Professional support, Perceived safety and Participation*. The questionnaire was found to discriminate between groups of women known to differ in their childbirth experience for example those with a shorter duration of labour had a significantly higher score on all scales than those with a longer duration of labour. This questionnaire has not been validated in the UK. When a health measurement tool is translated, it must be translated well linguistically but also adapted culturally to maintain its validity. The process of translation is well described [2]. When a questionnaire has been translated it is important to establish if the content validity has been preserved in the new translation. In the translation process of the CEQ, two independent professional translators, a native English translator and the other a native Swedish translator specialised in health care, translated the questionnaire and gave their comments on the items. The Swedish research group compiled these two translations into one. This compiled version was back-translated by a native Swedish speaking colleague familiar with childbirth care.

Measuring the impact of an intervention on a woman’s childbirth experience is arguably as important as measuring its impact on outcomes such as caesarean delivery and perinatal morbidity or mortality and yet surprisingly it is rarely done [3]. None of the large international obstetric randomised controlled trials in the last decade have included maternal satisfaction as a reported secondary outcome. Dissatisfaction with the childbirth experience has been associated with a negative impact on breast feeding and infant bonding and an increase in postpartum depression, post-traumatic stress disorders, future terminations of pregnancy and preference for caesarean delivery in future pregnancies [4].

The lack of a robust validated tool for evaluating labour experience in the UK is a topical issue in the UK at present. Indeed the National Institute for Health and Care Excellence (NICE) say ‘Women’s experiences of birth vary enormously and are influenced by many factors… Most studies investigated the effectiveness of any interventions used during birth, but insufficiently reported women’s psychological and emotional wellbeing and their birth experiences’. The findings consistently showed that measurement of these factors was not robustly undertaken. A standardised method to measure and quantify women’s psychological and emotional wellbeing and their birth experiences is urgently required to support any study investigating the effectiveness of interventions, techniques or strategies during birth [5].

Various instruments exist to measure childbirth experience. Two tools have been validated for use in the UK: the Perceptions of Care Adjective Checklist Revised (PCACL-R) and the Patient Perception Score (PPS) [6]. Both of which have their limitations. The Care Quality Commission Maternity Survey has been widely used across the UK to measure the quality of maternity services. In 2007 the Maternity Survey was the first national trust based survey of maternity care in the UK. The face validity of the 2007 Maternity Survey had been established using interviews with postnatal mothers and the survey was pilot tested in 2772 women in 2006 [7]. The pilot survey had a 59% response rate and items with a high non-response rate (>5.5%) were removed or changed. The 2007 Maternity Survey was completed by over 26,000 women who gave birth in February 2007 and had an identical response rate to the pilot study. The 2010 Maternity Survey was based on modifications of the 2007 survey; removing items with high non-response rates and high floor/ceiling effects and cognitive interviews with postnatal mothers to establish face validity of the revised questionnaire [8]. An example of one of the items in the 2010 Maternity Survey is given here: “During your labour and birth, did you feel you got the pain relief you wanted?” The Maternity Survey focuses on the practicalities of maternity care in the NHS for example: time waited for perineal suturing; whether skin to skin contact with the baby was provided after birth. While these practicalities are hugely important in measuring the care delivered they do not measure how a woman feels about her birth experience.

In the absence of a robust, validated tool for measuring birth experience in the UK, a plan was therefore made to validate the Childbirth Experience Questionnaire for content validity and construct validity in an English speaking population. It was also decided to measure test-retest validity and criterion validity for the CEQ, as this has not been done previously.

## Methods

### Participants and setting

A prospective postnatal postal questionnaire study of 350 women who laboured and gave birth to their first baby at 37–41 completed weeks at Nottingham University Hospitals NHS Trust (NUH) between October 2013 and July 2014 was performed. The CEQ was tested for face validity on 25 postnatal mothers.

Women were identified in the postnatal period by their obstetrician or midwife on the postnatal ward and offered an information sheet and entry to the study. Those who agreed to join signed a written consent form. Women were sent two questionnaires in the post when they were one month postnatal: the CEQ and Part C of the Care Quality Commission Maternity Survey [8]. Women were also sent the CEQ two weeks later. Women were given the option of completing the questionnaire via email or via an online survey tool instead of post. Reminders were sent after two weeks to those who had not replied to the original request.

Inclusion criteria were primiparity, a singleton live fetus, women that laboured (including women who required delivery by caesarean section during the latent phase of labour or for a failed induction of labour) at term (≥37^+0^ weeks) and women aged 18 years or older.

Exclusion criteria were women whose baby had died and women whose babies have been unexpectedly admitted to the Neonatal Intensive Care Unit or Special Care Baby Unit. Demographic data and basic data on their delivery outcome were collected from the hospital notes at hospital discharge.

### Childbirth experience questionnaire (CEQ)

The CEQ has 22 statements assessing four domains of childbirth experience (Table 1) [1]. For 19 of the items the response format is a 4-point Likert Scale and three of the items are assessed using a visual analogue scale (VAS). Higher scores indicate better childbirth experience.

**Table 1:** **Childbirth experience questionnaire (CEQ) domains and included items**

**Domain**	**Items**
*Own capacity*	Labour and birth went as I had expected
	I felt strong during labour and birth
	I felt capable during labour and birth
	I was tired during labour and birth
	I felt happy during labour and birth
	I felt that I handled the situation well
	As a whole, how painful did you feel childbirth was?*
	As a whole, how much control did you feel you had during childbirth?*
*Professional support*	My midwife devoted enough time to me
	My midwife devoted enough time to my partner
	My midwife kept me informed about what was happening during labour and birth
	My midwife understood my needs
	I felt very well cared for by my midwife
*Perceived safety*	I felt scared during labour and birth
	I have many positive memories from childbirth
	I have many negative memories from childbirth
	Some of my memories from childbirth make me feel depressed
	My impression of the team’s medical skills made me feel secure
	As a whole, how secure did you feel during childbirth?*
*Participation*	I felt I could have a say whether I could be up and about or lie down
	I felt I could have a say in deciding my birthing position
	I felt I could have a say in the choice of pain relief

*VAS-scale with anchors.

### Care quality commission maternity survey

The Maternity Survey 2010 is the best available ‘gold standard’ for measuring birth experience in the UK as it has been tested for face validity using interviews with postnatal mothers and has been pilot tested and improvements based on removal of high non-response items and items with high floor/ceiling effects have been made with each subsequent use (extensive pilot testing 2006, nationwide use 2007).

### Statistics and data analysis

The planned sample size was 220 women. This was based on the recommendation of a sample size of ten times the number of observed variables in the health measurement tool being evaluated [1,9]. The CEQ has 22 items therefore 220 completed questionnaires would be required. As it is common to get missing values rendering a returned questionnaire uninterpretable, the target was to receive 250 completed questionnaires to analyse. Assuming a 70% response rate (from that achieved in the original Swedish study), it was determined that it would be necessary to send the questionnaire out to 350 women to achieve a final sample size of 220 completed questionnaires.

### Face validity

A small subgroup of 25 postnatal women on the postnatal ward were asked to complete the CEQ and then asked some questions face to face on whether the questionnaire was easy to understand, easy to complete and acceptable to them.

### Internal consistency

Reliability of the CEQ in its translated form was measured by calculating Cronbach’s alpha for each of the subscales and for the total scale. As a general rule, a value of Cronbach’s alpha > 0.7 is generally regarded as satisfactory.

### Construct validity

Construct validity of the CEQ in its translated form was measured by using the method of known-groups validation using data collected on women’s delivery outcomes. Known-groups validation assesses the ability of the instrument to distinguish between subgroups known to differ on key sociodemographic or clinical variables. A comparison was made of CEQ subscale scores and total CEQ score (average of the 4 individual subscale scores) for women with labour duration more than 12 hours versus less than 12 hours, women with oxytocin augmentation during labour versus without, women who had a spontaneous vaginal delivery versus those who had an operative delivery. These were the same comparative groups used to establish construct validity of the CEQ in Swedish women [1] and previous research has indicated that oxytocin augmentation [10], a long duration of labour [11,12] and operative birth have a negative impact on birth experience [10]. As the scale scores were not normally distributed a Mann Whitney *U* test was used to compare scale scores between the groups. Where there were a few missing items, the half-scale method was used so that when the respondent had answered at least half of the items in the scale the sum of the scores were divided by the number of answered items [9].

Effect sizes, as defined by Cohen, were computed as the difference between group mean scores divided by the pooled standard deviation of the two groups [13].

Effect sizes of 0.2-0.5 was regarded as “small”, 0.5-0.8 as “moderate” and above 0.8 as “large” [9].

### Test retest reliability

The results of the scored CEQ sent out at four and six weeks were compared. The two scores for each item on the questionnaire obtained two weeks apart were used to calculate the absolute score difference for each of the 22 items on the questionnaire.

This was used to calculate the index of agreement. As the items measure ordered categorical data, the quadratic weighted index of agreement (weighted kappa) was used as a measure of the correlation of the CEQ scores recorded at two week time intervals [9].

The methodology for test-retest reliability is described in full in a comprehensive text on quality of life assessment tools by Fayers and Machin [9]. In brief, when assessing test-retest reliability or ‘repeatability reliability’ one measures the proportion of agreement when the same instrument is applied on two occasions. The proportion of agreements is equal to the number of patients who respond in the same way in both assessments for example ‘yes’ to both assessments, or ‘no’ to both, divided by the total number of patients assessed. This gives a value of p_Agree_.

As some of the agreement between the two assessments would be expected to arise by chance, a kappa coefficient (κ) is used to assess the extent to which chance agreements impact on the overall proportion of agreement. p_Agree_ – p_Chance_ extracts the level of agreement that arises by chance alone. This leads to a kappa index of agreement which is scaled to a maximum value of 1. If κ equals 1 there is perfect agreement, if κ equals 0, the agreement is no better than that which would have arisen out of chance if the assessments were completed at random. When ordered categorical data is used such as a Likert scale in the case of the CEQ the authors recommend the use of a weighted form of κ (κ_Weight_) which takes into account that if a participant chooses a response 1 “very poor” on the first assessment and 5 “very good” on the second assessment this has a greater weight or greater degree of disagreement than if they were to choose 1 “very poor” on the first assessment and 2 “poor” on the second. κ_Weight_ is described by the authors as analogous to the intraclass correlation coefficient.

### Criterion validity

The 2010 Maternity Survey was selected as the best available comparator to measure criterion validity of the CEQ. Prior to analysis it was agreed that should measurements of content and criterion validity demonstrate that the CEQ was not a valid instrument then evidence of criterion validity would be interpreted with caution and not in isolation provide conclusive evidence of the CEQ’s validity. The results of the CEQ were scored according to the author’s instructions. The results of the Maternity Survey were scored according to the Care Quality Commission’s instructions. Criterion validity was measured by calculating the Pearson correlation coefficient for the CEQ and Maternity Survey scores.

All analyses were conducted using Stata Version 11.

Permission to conduct the study was obtained from the West of Scotland Research Ethics Service on 10th September 2013 (Reference 13/WS/0189).

## Results

A total of 355 eligible women were identified and of those 350 women agreed to join the study between 18th October 2013 and 3rd July 2014 and were sent postnatal questionnaires. Reminders were sent after two weeks to those who had not replied to the original request. Completed questionnaires were returned by 206 women (59% response rate). Of those 206 women, all completed the CEQ and 204 women completed the Maternity Survey. All 206 women were sent the CEQ two weeks later and 132 women returned the 2nd CEQ (64% response rate). Characteristics of the study population are shown in Table 2. Birth statistics for NUH in June 2014 are given for comparison with the study population and the percentages in the study population are similar for mode of delivery and onset of labour. Table 3 shows the number of missing items for each of the questionnaires. In general there was very little missing data. The data for two participants with high numbers of missing items (10 items missing in the 1st CEQ, 6 items missing in the 2nd CEQ) were excluded from the analyses.

**Table 2:** **Characteristics of the study population, n = 206 and characteristics of a sample of women who delivered in one selected month at the participating hospital for comparison**

	**Study population**	**June 2014 NUH statistics**
Maternal age, years, mean (SD)	29 (5.4)	
Gestational age, weeks, mean (SD)	39 (1.3)	
Normal vaginal delivery, number (%)	100 (49)	78 (46)
Operative delivery, number (%)	106 (51)	90 (54)
*Instrumental delivery*	*66 (32)*	*52 (31)*
*Caesarean delivery*	*40 (19)*	*38 (23)*
Onset of labour, number (%)		
*Spontaneous*	132 (64)	105 (62)
*Induced*	74 (36)	63 (38)
Labour duration more than 12 hours,* number (%)	43 (21)	
Oxytocin augmentation during labour, number (%)	107 (52)	
Neonatal Intensive Care Admission, number (%)	6 (3)	
Method used to return questionnaire, number (%)		
*Postal*	*133 (65)*	
*Email*	*73 (35)*	

*Labour duration could not be calculated for 4 women who had a caesarean section prior to the onset of active labour.

**Table 3:** **Number of missing items for each of the three questionnaires which were sent during the course of the study**

**Number of missing items**	**1st CEQ**	**Maternity survey**	**2nd CEQ**
0 items missing	202	150	121
1 item missing	2	40	8
2 items missing	0	14	1
3 items missing	1	2	0
6 items	0	0	1
10 items missing	1	0	0
16 items missing	0	0	1
**Total participants**	**206**	**204**	**132**

### Face validity

Twenty five women on the postnatal ward were asked to read the Childbirth Experience Questionnaire and were then asked questions about it. All women found the questionnaire easy to understand and complete. No respondents felt that any questions should be removed or found any of the questions were upsetting or offensive.

### Internal consistency

Cronbach’s alpha was ≥ 0.70 for all of the subscales (*Own capacity* 0.79, *Perceived safety* 0.83, *Participation* 0.72 and *Professional support* 0.94) (Table 4). Cronbach’s alpha for the total scale was 0.90. Cronbach’s alpha for all of the subscales in the original Swedish study validating the CEQ in a Swedish population are given for comparison.

**Table 4:** **Cronbach’s alpha for the domains of the CEQ and for the overall scale**

**Domain**	**Number of items**	**Cronbach’s alpha**	**Cronbach’s alpha from original Swedish study**
*Own capacity*	8	0.79	0.82
*Professional support*	5	0.94	0.88
*Perceived safety*	6	0.83	0.78
*Participation*	3	0.72	0.62
Total scale	22	0.90	

### Construct validity

Construct validity of the CEQ was measured using the methods of known-groups validation as shown in Table 5. Women with a shorter duration of labour were significantly more likely to have higher scores for subscales of the CEQ of *Own capacity* and *Perceived safety* and for the overall mean CEQ score than women with a longer duration of labour. There was no statistically significant difference between scores for the subscale of *Participation* or *Professional support* in women with a shorter duration of labour than a longer duration. These results were very similar for women who required oxytocin augmentation versus no oxytocin augmentation: statistically significantly higher subscale scores for *Own capacity* and *Perceived safety* and overall mean CEQ score, no statistically significant differences in scores for *Participation* and *Professional support*.

**Table 5:** **Differences in subscale scores and overall score of the CEQ by different groups**

	***Own capacity***	***Professional support***	***Perceived safety***	***Participation***	**Mean CEQ score**
Labour duration ≤ 12 hours	2.58 (0.55)	3.51 (0.68)	3.00 (0.68)	3.02 (0.84)	3.02 (0.52)
Labour duration > 12 hours	2.23 (0.51)	3.55 (0.51)	2.67 (0.68)	2.97 (0.57)	2.86 (0.44)
Unadjusted p-value	<0.001	0.54	0.004	0.45	0.011
Effect size	0.62	−0.06	0.48	0.06	0.31
No oxytocin augmentation	2.65 (0.56)	3.49 (0.64)	3.12 (0.60)	3.02 (0.83)	3.07 (0.49)
Oxytocin augmentation	2.35 (0.54)	3.53 (0.68)	2.74 (0.72)	2.98 (0.78)	2.90 (0.51)
Unadjusted p-value	<0.001	0.18	<0.001	0.61	0.014
Effect size	0.53	−0.06	0.55	0.05	0.33
Spontaneous vaginal delivery	2.64 (0.57)	3.55 (0.63)	3.10 (0.65)	3.15 (0.86)	3.11 (0.52)
Operative delivery	2.35 (0.53)	3.47 (0.69)	2.76 (0.69)	2.85 (0.73)	2.86 (0.47)
Unadjusted p-value	<0.001	0.53	<0.001	0.002	<0.001
Effect size	0.51	0.12	0.49	0.38	0.49

Data presented as mean (SD). Mann–Whitney *U*-test was used to compute p-values. Operative delivery includes instrumental vaginal and caesarean deliveries. Total score for the CEQ is the mean score of the 4 subscales. Numbers given to 2 significant figures.

Women who had a vaginal delivery were significantly more likely to have higher scores for all subscales of the CEQ (with the exception of the subscale *Professional support)* and for the overall mean CEQ score.

### Test-retest reliability

There were 132 participants who completed both the first and second CEQ. One woman had incomplete data rendering the first CEQ unusable and one patient had incomplete data rendering the second CEQ unusable. For the remaining 130 participants the quadratic weighted index of agreement (weighted Kappa) for each subscale of the CEQ and for the overall CEQ score was calculated and are presented in Table 6.

**Table 6:** **Test-retest reliability for the CEQ; the quadratic weighted index of agreement (weighted kappa) is given**

**CEQ subscale**	**Observed Agreement**	**Expected agreement**	**Weighted kappa**	**P Value**
*Own capacity*	90%	70%	0.66	<0.001
*Professional support*	90%	70%	0.69	<0.001
*Perceived safety*	90%	73%	0.64	<0.001
*Participation*	89%	73%	0.60	<0.001
Overall CEQ score	91%	70%	0.68	<0.001

A value of weighted Kappa between 0.61 – 0.80 represents substantial agreement between the two scores [14]. All subscales of the CEQ except *Participation* were found to have substantial agreement between the scores obtained at the completion of the CEQ at 4 weeks postnatal and its completion at 6 weeks postnatal. The subscale *Participation* was found to have moderate agreement between the two sets of scores. Overall the CEQ may be considered a very reliable instrument when used on separate occasions.

### Criterion validity

Criterion validity was measured by calculating the Pearson correlation coefficient for the CEQ and Maternity Survey scores as shown in Table 7. The correlation coefficient indicates the degree of linear relationship between two variables. A correlation coefficient value ≤ 0.35 is generally considered to represent a weak correlation, 0.36 to 0.67 to represent a moderate correlation and 0.68 to 1.0 to represent a strong correlation with coefficients ≥ 0.90 representing very strong correlations [15]. All subscales had moderate correlations and the total scale had a strong correlation with the Maternity Survey.

**Table 7:** **Correlation between CEQ subscale scores and overall maternity survey scores**

**CEQ subscale**	Pearson correlation coefficient*
*Own capacity*	R = 0.50
*Professional support*	R = 0.67
*Perceived safety*	R = 0.61
*Participation*	R = 0.42
Overall mean CEQ score	R = 0.73

*Pearson correlation coefficient was calculated using Stata V11.

## Discussion

### Statement of principal findings

Face validity of the CEQ in a UK population was demonstrated with all respondents reporting that the questionnaire was easy to understand and complete. A statistically significant higher CEQ score for subgroups of women known to report a better birth outcome (shorter labour, no oxytocin augmentation and vaginal delivery) demonstrated construct validity of the CEQ in 2 subscales. A weighted kappa of 0.68 for the full scale demonstrated test-retest reliability of the CEQ. A Pearson correlation co-efficient of 0.73 demonstrated a strong correlation between the results of the CEQ (total score) and the results of the ‘gold standard’ assessment of childbirth experience in the UK: the Maternity Survey and hence criterion validity of the CEQ.

### Strengths and weaknesses of the study

Unfortunately a lower response rate (70% predicted, 59% achieved) than anticipated meant that the final sample size was 206, 6% lower than intended (original sample size 220).

Small effect sizes were seen for the subscale *Perceived safety* and for the overall total CEQ score. The total score for the CEQ has not previously been used as an overall measure of childbirth experience but this study demonstrates that the total score has good test-retest reliability, good correlation with the ‘gold standard’ Maternity Survey and significant differences (though with small to moderate effect sizes) between groups known to differ in their childbirth experience.

### Strengths and weaknesses in relation to other studies

When the developers of the CEQ sought to validate the Swedish version of the questionnaire using the method of known-groups validation they found that women with long labours, oxytocin augmentation and operative delivery had significantly lower scores for all subscales of the CEQ. Interestingly in this study, UK women with longer labours and oxytocin augmentation had no statistically significant differences in their scores for *Professional support* than women with shorter labours and no oxytocin augmentation. Dencker *et al.* did comment that the weakest effect sizes observed were seen with the *Professional support* domain [1]. It is plausible that women who experience the most intervention in labour may well feel that they have more input from staff and therefore perceive a higher level of professional support, due to the time spent counselling for and administering the intervention. Certainly women who experience continuous support in labour are less likely to be dissatisfied with their birth experience (RR 0.73, 95% CI 0.65-0.83) [16] However the 2013 Maternity Survey results found that women who had a normal vaginal delivery were less likely to report being ‘left alone by staff’ at a time that worried them than women who had an assisted birth during labour [17] and an observational study of the quantity of ‘supportive care’ that women received from their midwife showed that although nulliparous women received larger quantities than multiparous women, women who had an epidural had the same amount of supportive care as those without [18].

The effect sizes seen for *Own capacity* and *Perceived safety* were very similar to those observed in the Swedish validation study. The effect sizes seen for *Professional Support* and *Participation* were smaller than those observed in the Swedish validation study. This difference may well be due to the differences in characteristics of the participants included in the two studies; the Swedish study only included women in established spontaneous labour (≥4 cm cervical dilatation) and had a 5% caesarean section rate (compared to 19% in our study population). Given that the greatest effect sizes were observed in comparing women who experienced a vaginal delivery to those who had a caesarean section and that a caesarean section for women in the Swedish study may well have been far less anticipated than in our study, and therefore have a greater impact on childbirth experience this could explain partly the differences in magnitude of effect sizes observed.

The need for a ‘robust method of assessing women’s satisfaction with their birth experience’ was previously identified by the National Institute for Health and Care Excellence as one of 3 main research priorities for improving intrapartum care [5]. In a review of the merit of existing questionnaires measuring birth satisfaction, (which did not include the CEQ), the authors recommend the use of the Intrapartal-Specific Quality from the Patient’s Perspective questionnaire (QPP-I) [19]. This is a Swedish questionnaire which has not been validated in a UK population [20]. Other questionnaires which have been developed and validated in the UK include The Perceptions of Care Adjective Checklist Revised (PCACL-R) [21] and the Patient Perception Score (PPS) [6]. Arguably these two questionnaires have yet to be proven valid and reliable measures of childbirth experience.

In the case of the Perceptions of Care Adjective Checklist Revised (PCACL-R) [21]: construct validity was reportedly established in a number of ways: by comparison with the results from a single question about satisfaction: ‘*How well do you feel staff communicated with each other about your care in labour and delivery*?’, by scores for the Index of Multiple Deprivation, by length of labour and by mode of delivery. The authors postulated that there would be no difference in birth experience based on labour length and considered it a measure of validity that there was no correlation between labour length and PCACL-R scores. The authors predicted that women who experienced a vaginal birth would have higher satisfaction scores than those who had experienced an operative delivery but in fact found that women who had a vaginal birth had lower scores of satisfaction when compared to those women who had an operative birth. The authors conclude that construct validity for the instrument was ‘good’ despite these shortcomings. Content validity, criterion validity and test-retest reliability were not measured.

The Patient Perception Score (PPS) [6] consists of three questions rated using a 5-point Likert scale: “I felt I was treated with respect by the doctor(s)”; “I felt safe at all times”; “I felt well informed due to good communication with the doctor(s)”. It was validated by its authors in a sample of 150 women who had undergone operative delivery under regional anaesthesia (98%) within the preceding 24 hours. Their intention was to validate a short, practical tool that could evaluate women’s satisfaction with their experience of an operative delivery. Siassikos *et al.* suggest that as a tool to measure communication during an operative delivery the PPS is a useful and valid tool, but they do not state that the tool covers all aspects of labour and birth which impact on the multidimensional nature of maternal satisfaction.

This study supports the use of the CEQ in its translated form as ‘robust method of assessing women’s satisfaction with her birth experience’ and can be used as valid and reliable tool to measure childbirth experience in the UK population [5]. This study demonstrates new evidence that the total CEQ score may be used to measure childbirth experience.

### Unanswered questions and future research

A new version of the CEQ (CEQ 2) comprising of 25 items has been developed and is currently being validated in Sweden. This work is being done to develop the domains of ‘Professional Support’ and ‘Participation’. The domain for ‘Professional Support’ was demonstrating high ceiling effects and the authors decided that the domain for ‘Participation’ required more items on decision-making. Eight questions from the CEQ have been removed (comprising the 2 domains of ‘Professional Support’ and ‘Participation’) and 11 new questions have been added. Of the 11 new items, some items have simply been reworded to try to capture more negative experiences and some items are completely new. If this new version of the CEQ is proven to be an improvement on the original CEQ then it will require validating in a UK population.

## Conclusions

This study has looked extensively at the validity and reliability of the CEQ in a UK population: by comparing its results to the nationally used childbirth survey, the ‘Maternity Survey’; by comparing the scores for women known to differ in their childbirth experience; by comparing the scores when the questionnaire is completed by the same participant on two separate occasions and by collecting the views of women who have completed the questionnaire under the watchful eye of a researcher.

This study demonstrates that the Childbirth Experience Questionnaire is a valid and reliable measure of childbirth experience in the UK population.

## Abbreviations

CEQ: Childbirth Experience Questionnaire
NICE: National Institute for Health and Care Excellence
PCACL-R: Perceptions of Care Adjective Checklist Revised
PPS: Patient Perception Score
CQC: Care Quality Commission
NUH: Nottingham University Hospitals NHS Trust
UK: United Kingdom
